# Longitudinal trends in community-onset bacteraemia caused by ceftriaxone non-susceptible *Escherichia coli, Proteus mirabilis, Klebsiella oxytoca*, and *Klebsiella pneumoniae* (2016–2024)

**DOI:** 10.1093/jacamr/dlag014

**Published:** 2026-02-13

**Authors:** Raveena Patel, Laura G Stoudenmire, Cong Cheng, Xianyan Chen, Bryan P White, Daniel B Chastain

**Affiliations:** Department of Pharmacy, Phoebe Putney Memorial Hospital, Albany, GA 31701, USA; Department of Pharmacy, Phoebe Putney Memorial Hospital, Albany, GA 31701, USA; Department of Statistics, University of Georgia, Franklin College of Arts and Sciences, Athens, GA 30602, USA; Department of Epidemiology & Biostatistics, University of Georgia College of Public Health, Athens, GA 30602, USA; Department of Pharmacy, OU Health Medical Center, Oklahoma City, OK 73104, USA; Department of Clinical and Administrative Pharmacy, University of Georgia College of Pharmacy, Albany, GA 31701, USA

## Abstract

**Background:**

The increasing prevalence of ESBL-producing Enterobacterales complicates treatment and worsens outcomes. However, contemporary data describing the prevalence and temporal trends in established risk factors for community-onset ceftriaxone non-susceptible (CRO-NS) bacteraemia remain limited.

**Methods:**

This retrospective cohort study included adults with community-onset bacteraemia due to *Escherichia coli,* , *Klebsiella pneumoniae*, *Klebsiella oxytoca*, or *Proteus mirabilis,* evaluated at Phoebe Putney Health System (Albany, GA) between January 2016 and November 2024. Community-onset was defined by the first positive blood culture collected in the ER or ≤48 h of presentation. CRO susceptibility was determined by institutional antimicrobial susceptibility testing. Polymicrobial bacteraemia, incomplete records, or interfacility transfer were excluded.

**Results:**

Among 1392 patients with community-onset bacteraemia, 127 (9.1%) were due to CRO-NS isolates, with *E. coli* accounting for 83%. The prevalence of CRO-NS isolates doubled over the study period, increasing from 7% in 2016 to 16% in 2024 (*P* = 0.008), reflecting an approximate annual increase of 1%. Most patients were older adults (median age 67), female (57%), and Black or African American (53%), with multiple comorbidities (median Charlson Comorbidity Index 5). A urinary source was most common (67%). Recent antibiotic use (≤30 days: 37%; ≤90 days: 61%) and prior hospitalization (≤90 days: 39%; ≤1 year: 61%) were frequent. Most established risk factors remained stable over time.

**Conclusion:**

Community-onset CRO-NS bacteraemia more than doubled over nine years, driven primarily by *E. coli*. These findings demonstrate the expanding burden of antimicrobial resistance in the community and emphasize the importance of local surveillance and risk-based empiric prescribing strategies.

## Background

Antimicrobial resistance (AMR) remains one of the foremost global health threats, with rising incidence driven by both healthcare-associated and community-acquired infections. Among the most clinically significant contributors to AMR are Enterobacterales, a diverse order of Gram-negative bacteria that includes *Escherichia coli*, *Klebsiella pneumoniae*, *Klebsiella oxytoca*, and *Proteus mirabilis.*^1–3^ These organisms frequently produce extended-spectrum β-lactamases (ESBLs), enzymes capable of hydrolysing penicillins, third-generation cephalosporins, and aztreonam, thereby severely limiting treatment options.^1,4–6^

Although ESBL-producing Enterobacterales were initially considered primarily healthcare-associated pathogens, their presence in the community has increased substantially over the past two decades.^7,8^ In the USA, the Centres for Disease Control and Prevention (CDC) reported a 7% increase in community-onset ESBL infections from 2019 to 2020, a trend further exacerbated by the COVID-19 pandemic, which contributed to increased healthcare exposure and inappropriate antimicrobial prescribing.^9^

ESBL-producing Enterobacterales bacteraemia is associated with significant morbidity and mortality, with reported mortality rates ranging from 12% to 41%.^10–12^ Optimizing empiric antimicrobial therapy is critical to improving outcomes. However, prior carbapenem exposure, the preferred treatment for ESBL infections, has been associated with a three- to five-fold increased risk of subsequent carbapenem-resistant Enterobacterales (CRE), reflecting selective antimicrobial pressure that favours the emergence and propagation of resistant organisms.^13–15^ Therefore, accurate identification of individuals at increased risk for ESBL-producing Enterobacterales bacteraemia is critical to guide empiric therapy while preserving antimicrobial stewardship efforts.

Several risk factors for ESBL-producing Enterobacterales bacteraemia have been previously established, including advanced age, comorbid conditions, prior healthcare exposure, invasive devices, recent antibiotic use, and select medical procedures.^8,11,16–20^ However, there is limited longitudinal data characterizing how the prevalence of these risk factors has evolved in parallel with increasing rates of community-onset ESBL-producing Enterobacterales bacteraemia in the USA.

Given challenges with routine phenotypic ESBL testing, ceftriaxone non-susceptibility (CRO-NS) is commonly used as a pragmatic surrogate for ESBL-associated resistance consistent with clinical trials and guideline recommendations.^6,21,22^ The objectives of this study were to (i) describe longitudinal trends in community-onset ceftriaxone non-susceptible (CRO-NS) *E. coli*, *K. pneumoniae*, *K. oxytoca*, and *P. mirabilis* bacteraemia from 2016–2024 and (ii) conduct a restricted exploratory multivariable analysis to assess independent associations between core demographic characteristics and CRO-NS bacteraemia. These findings aim to enhance clinical awareness of evolving epidemiologic patterns and provide insight into changing risk profiles.

## Methods

### Study design and setting

We conducted a retrospective observational cohort study to characterize trends in community-onset bacteraemia caused by CRO-NS *E. coli*, *K. pneumoniae*, *K. oxytoca*, and *P. mirabilis* within the Phoebe Putney Health System, a regional healthcare network in southwest Georgia (USA), from January 1, 2016, to November 30, 2024.

### Study population

We identified adults (≥18 years) with at least one blood culture positive for *E. coli*, *K. pneumoniae*, *K. oxytoca*, or *P. mirabilis*, obtained in the emergency department or within 48 hours of hospital presentation, consistent with the definition of community-onset bacteraemia.^23^ Only cases with CRO-NS isolates were included, as determined by institutional antimicrobial susceptibility testing (AST) based on Clinical and Laboratory Standards Institute (CLSI) breakpoints.

Patients with polymicrobial infections, those transferred from outside facilities, or those with incomplete medical records were excluded. To minimize selection bias, only the first isolate per patient per calendar year was included. However, patients were eligible for re-inclusion in subsequent years if they experienced recurrent bacteraemia episodes, regardless of the interval between episodes.

### Data collection

De-identified patient data were extracted from the electronic medical record (EMR) and entered into REDCap, a secure, web-based data capture platform hosted by the University of Georgia.^24,25^

Collected variables included patient demographics (age, sex, race, and place of residence prior to presentation), clinical characteristics, comorbidities, prior healthcare exposures, medication use, and infection severity at presentation. Residence was categorized as home, long-term care facility, or other institutional settings (such as shelters, rehabilitation centres, or correctional facilities).

Clinical characteristics included the source of bacteraemia based on provider documentation, history of ESBL infection within one year, recent gastrointestinal or urologic procedures (within 30 days), and injection drug use.^16^ Comorbid conditions were assessed using the Charlson Comorbidity Index.^26^

Medication exposures were evaluated for systemic glucocorticoids, proton pump inhibitors, chemotherapy (within 90 days), and antibiotic use (within 30 and 90 days). Healthcare exposures included hospitalizations (within 90 days or one year).

### Outcomes

The primary outcomes were to (i) assess annual trends in community-onset CRO-NS *E. coli*, *K. pneumoniae*, *K. oxytoca*, and *P. mirabilis* bacteraemia from 2016 to 2024 and (ii) evaluate temporal changes in the prevalence of established risk factors among this population. The study specifically evaluated changes in the distribution of known risk factors rather than identifying new predictors of CRO-NS or ESBL-producing Enterobacterales bacteraemia. Secondary analyses compared demographics, comorbidities, and healthcare exposures before versus after the onset of the COVID-19 pandemic, defined by the first reported USA case on January 21, 2020.

### Statistical analysis

Descriptive statistics were used to summarize the prevalence of CRO-NS Enterobacterales bacteraemia, patient characteristics, and clinical variables. Categorical variables were reported as frequencies with percentages, while continuous variables were expressed as medians with interquartile ranges (IQR).

Annual prevalence estimates of CRO-NS Enterobacterales bacteraemia and associated risk factors were calculated to assess temporal trends. Linear regression analysis was used to estimate annual changes in CRO-NS Enterobacterales bacteraemia prevalence. Pre-versus post-COVID-19 comparisons were performed using chi-square or Fisher’s exact tests for categorical variables and *t*-tests or Mann–Whitney *U* tests for continuous variables, based on distribution.

Multivariable weighted logistic regression was performed to evaluate factors independently associated with community-onset CRO-NS *E. coli*, *K. pneumoniae*, *K. oxytoca*, and *P. mirabilis* bacteraemia. Covariates included sex, age >65 years, race (Black or African American vs non-Black or African American), and year of blood culture collection. These variables were selected *a priori* based on existing literature describing established demographic predictors of CRO-NS or ESBL-producing Enterobacterales bacteraemia, as well as data feasibility within our dataset.^8,27^ Selection was designed to maintain consistency with previously published risk factor analyses and to ensure that all covariates were available for both CRO-NS and susceptible comparator groups.

Because detailed clinical exposure data were only available for patients with CRO-NS bacteraemia and were not collected for patients with ceftriaxone-susceptible isolates, these variables were not eligible for inclusion in comparative modelling. As a result, regression analyses were restricted to demographic variables available across the entire cohort.

Age was dichotomized at >65 years, a standard and clinically meaningful threshold commonly applied in infectious diseases outcomes research.^28,29^ Although the median age of the cohort was 67 years, use of this *a priori* cut-off ensured comparability with prior studies and alignment with established epidemiologic frameworks rather than deriving a data-dependent threshold specific to the present population.

Regression coefficients, 95% confidence intervals (CIs), and *P*values were reported. All statistical analyses were performed using R software (R Foundation for Statistical Computing, Vienna, Austria), and a two-sided *P* value <0.05 was considered statistically significant.

### Ethical considerations

This study was approved by the University of Georgia Institutional Review Board (PROJECT00010366), with a waiver of HIPAA authorization and informed consent due to the retrospective study design and use of de-identified data.

## Results

A total of 1392 cases of community-onset bacteraemia due to *E. coli*, *K. pneumoniae*, *K. oxytoca*, or *P. mirabilis* were identified between 2016 and 2024. Of these, 127 (9.1%) were caused by CRO-NS isolates. The annual number of CRO-NS bloodstream infections increased over the study period, rising from nine cases in 2016 to a peak of 22 cases in 2024.

*E. coli* was the predominant pathogen, accounting for 83.4% (106/127) of ceftriaxone non-susceptible cases, followed by *K. oxytoca* (11.8%, 15/127), *K. pneumoniae* (3.1%, 4/127), and *P. mirabilis* (1.6%, 2/127).

**Figure 1.**
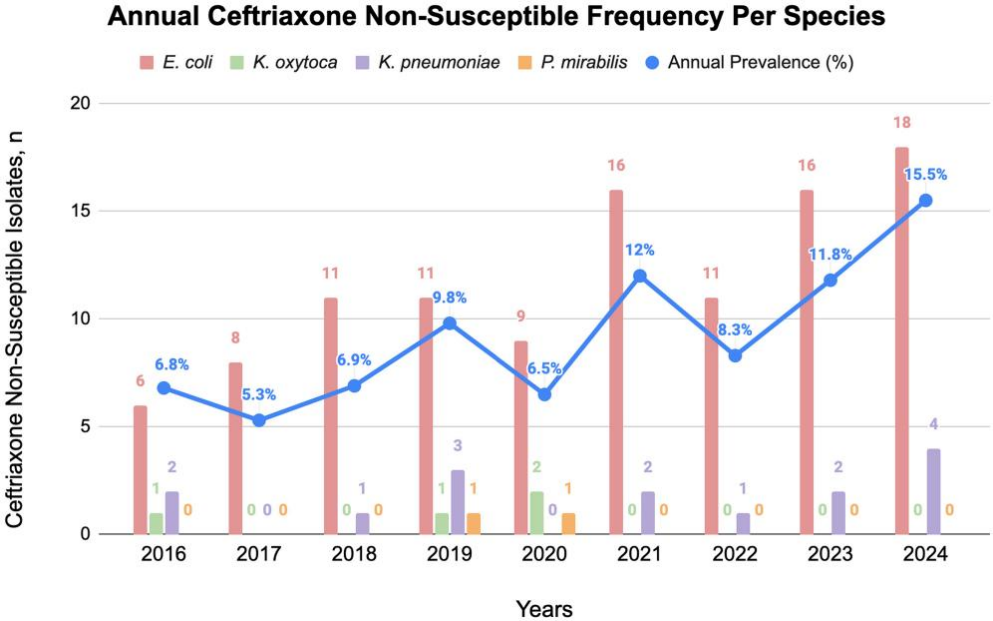
Annual frequency of ceftriaxone non-susceptibility among four bacterial species (*Escherichia coli, Klebsiella oxytoca, Klebsiella pneumoniae,* and *Proteus mirabilis*) from 2016 to 2024. Prevalence was calculated annually as the proportion of ceftriaxone non-susceptible isolates relative to the total number of isolates (ceftriaxone non-susceptible and ceftriaxone susceptible) for each species. The trendline represents the overall annual prevalence of ceftriaxone non-susceptible isolates aggregated across all four species. Annual prevalence is calculated as the ratio of ceftriaxone non-susceptible isolates to the total isolates per year: 2016 (9/132), 2017 (8/150), 2018 (12/174), 2019 (16/163), 2020 (12/184), 2021 (18/150), 2022 (12/144), 2023 (18/153), 2024 (22/142). See Table S1 for the prevalence of each species per year.

The overall prevalence of CRO-NS Enterobacterales bacteraemia significantly increased from 6.8% in 2016 to 15.5% in 2024 (*P* = 0.008), reflecting an estimated annual increase of approximately 1% (Figure 1, Table S1 (available as Supplementary data at *JAC-AMR* Online)). This trend was primarily driven by *E*. *coli*, rising from 6.2% in 2016 to 21.2% in 2024 (*P* = 0.002), corresponding to an annual increase of 1.64%. In contrast, the prevalence of *K. pneumoniae* decreased slightly (−2.16% per year, *P* = 0.550), *K. oxytoca* increased modestly (1.13% per year, *P* = 0.608), and *P. mirabilis* prevalence remained stable (−0.11% per year, *P* = 0.825).

The median age of patients was 67 years (IQR 58–77), with annual variability but no consistent temporal trend (Table 1, Table S2). Female patients accounted for 57% of cases, with proportions varying by year (range 42%–78%). Over half of patients (53%) identified as Black or African American, compared to 43% White, with no sustained change over time.

**Table 1.** Baseline characteristics of adults with community-onset ceftriaxone non-susceptible *E. coli, K. pneumoniae, K. oxytoca, and P. mirabilis* bacteraemia

Characteristic	Overall (*n* = 127)	Annual change	*P* value
Age (years), median (IQR)	67 (58, 77)	−0.05	0.946
Female sex	57%	−0.73%	0.718
Race			
White	43%	−0.49%	0.619
Black or African American	53%	−0.37%	0.722
Other	4%	8.56%	0.157
Admitting source			
Home	77%	1.05%	0.397
Long term care	20%	−1.98%	0.181
Other*^a^*	3%	0.93%	0.223
Infection source			
Urinary tract	67%	1.85%	0.449
Respiratory tract	2%	0.48%	0.197
Skin and soft tissue	6%	−0.58%	0.523
Gastrointestinal tract	12%	0.17%	0.888
Unknown*^b^*	13%	−1.93%	0.099
History of ESBL within 1 year, any source	14%	−1.43%	0.446
Comorbid conditions and medical history			
Gastrointestinal procedures within 30 days	6%	0.40%	0.668
Urological procedures within 30 days	6%	−0.69%	0.579
Injection drug use	<1%	—	—
Congestive heart failure	26%	−0.07%	0.954
Chronic pulmonary disease	17%	0.13%	0.845
Diabetes	43%	0.23%	0.891
Moderate or severe renal disease	11%	−0.68%	0.544
Moderate or severe liver disease	6%	1.40%	0.045
Metastatic solid tumour	6%	0.95%	0.290
Solid tumour (non-metastatic)	16%	−2.77%	0.103
HIV	2%	0.67%	0.035
Charleson Comorbidity Index, median (IQR)	5(3, 7)	0.08	0.652
Medication use within 90 days			
Glucocorticoids	18%	−0.27%	0.838
Proton pump inhibitors	38%	−2.47%	0.119
Chemotherapy	11%	1.34%	0.095
Antibiotic use within 30 days*^c^*	37%	2.98%	0.044
Anti-pseudomonal*^d^*	36%	−0.48%	0.909
Anti-MRSA*^e^*	25%	0.69%	0.876
Cephalosporin*^f^*	38%	−0.90%	0.845
Fluoroquinolone	32%	1.46%	0.631
Penicillins*^g^*	11%	1.88%	0.206
Carbapenems	4%	1.73%	0.262
Other*^h^*	32%	1.67%	0.613
Antibiotic use within 90 days*^c^*	61%	0.47%	0.830
Anti-pseudomonal*^d^*	62%	−1.25%	0.685
Anti-MRSA*^e^*	37%	−2.26%	0.496
Cephalosporin*^f^*	56%	3.13%	0.341
Fluoroquinolone	45%	−0.52%	0.873
Penicillins*^g^*	10%	2.70%	0.033
Carbapenems	10%	0.19%	0.907
Other*^h^*	31%	4.45%	0.066
Hospitalization within 90 days	39%	−1.08%	0.687
Hospitalization within 1 year	61%	0.45%	0.875

^*a*^Defined as being admitted from shelters, rehabilitation centres, or correctional facility.

^*b*^Unclear source of infection or infection was due to multiple sources.

^*c*^Antibiotics may be classified under more than one pharmacological drug class.

^*d*^Anti-pseudomonal includes Cefepime, Pip-Tazo, Meropenem, Gentamicin, Ciprofloxacin, Levofloxacin.

^*e*^Anti-MRSA includes Vancomycin, TMP-SMX, Doxycycline, Clindamycin.

^*f*^Cephalosporin includes Cefazolin, Cefdinir, Cefepime, Cefoxitin, Ceftriaxone, Cephalexin.

^*g*^Penicillins includes Amoxicillin, Amox-Clav, Ampicillin, Amp-Sulb.

^*h*^Other includers Azithromycin, Clarithromycin, Metronidazole, Nitrofurantoin.

Most patients presented from home (77%), while 20% were admitted from long-term care facilities. The urinary tract was the most common source of infection (67%), and remained the predominant source throughout the study.

The median Charlson Comorbidity Index was 5 (IQR 3–7), indicating substantial comorbidity. Common conditions included diabetes (43%), congestive heart failure (26%), and chronic pulmonary disease (17%). A prior ESBL-producing infection within the preceding year was documented in 14% of patients but varied widely by year, ranging from 0% in 2019 to 42% in 2020, before declining to 5% in 2024. Recent urologic or gastrointestinal procedures were infrequent (∼6% each), and injection drug use was rare (<1%).

Antibiotic exposure prior to presentation was common, with 37% of patients receiving antibiotics within 30 days and 61% within 90 days. Notably, 30-day antibiotic use increased over time, reaching 55% in 2024. Recent hospitalizations were documented in 39% (within 90 days) and 61% (within one year) of patients.

Evaluation of temporal changes in established risk factors revealed that most did not significantly correlate with rising ceftriaxone non-susceptibility. However, the prevalence of moderate or severe liver disease (+1.40% per year, *P* = 0.045), HIV (+0.67% per year, *P* = 0.035), antibiotic use within 30 days (+2.98% per year, *P* = 0.044), and penicillin exposure within 90 days (+2.70% per year, *P* = 0.033) significantly increased over the study period.

Comparisons between the pre-COVID-19 and post-COVID-19 periods revealed no significant differences in baseline demographics or comorbidities (Table S3). Although 30-day antibiotic exposure was more common post-COVID (42.7% vs 26.7%), this difference was not statistically significant (*P* = 0.110). However, exposure to ‘other’ antibiotics (e.g. azithromycin, metronidazole, nitrofurantoin) within 90 days increased significantly from 14.8% pre-COVID to 39.2% post-COVID (*P* = 0.049).

To assess independent predictors of CRO-NS, we performed multivariable weighted logistic regression among 1392 patients with community-onset Enterobacterales bacteraemia. The model included 1265 patients with ceftriaxone-susceptible isolates and 127 with CRO-NS isolates and adjusted for sex, age >65 years, race (Black or African American vs non–Black or African American), and year of blood culture collection.

In adjusted analyses, Black or African American race was independently associated with a 25% lower likelihood of CRO-NS (AdjOR = 0.753, 95% CI 0.609–0.930, *P* = 0.008) (Table 2). Female sex (AdjOR = 0.826, 95% CI 0.669–1.021, *P* = 0.077) and age >65 years (AdjOR = 0.884, 95% CI 0.714–1.095, *P* = 0.259) were not significantly associated with CRO-NS. Year of collection was independently associated with increasing prevalence of CRO-NS (AdjOR = 1.127, 95% CI 1.081–1.176, *P*=<0.001), confirming a persistent upward trend over time.

**Table 2.** Weighted regression analysis for *E. coli, K. pneumoniae, K. oxytoca, and P. mirabilis*

	Adjusted odds ratio	95% Confidence interval	*P* value
Female vs male sex	0.826	(0.669, 1.021)	0.078
Age >65 years	0.884	(0.714, 1.095)	0.259
Black or African American vs non–Black or African American	0.753	(0.609, 0.930)	0.008
Collection year	1.127	(1.081, 1.176)	<0.001

## Discussion

In this 9-year longitudinal cohort study, we observed a significant and sustained increase in the prevalence of community-onset bacteraemia caused by CRO-NS *E. coli*, *K. pneumoniae*, *K. oxytoca*, or *P. mirabilis*. The proportion of CRO-NS bloodstream infections more than doubled, rising from 6.8% in 2016 to 15.5% in 2024, driven primarily by the increasing prevalence of *E. coli*. These findings reinforce the growing body of evidence demonstrating the expanding epidemiology of ESBL-producing Enterobacterales beyond healthcare settings, underscoring the escalating threat of AMR in community-associated infections.^9,23^

Our results align with national and global studies reporting a steady rise in ESBL-producing Enterobacterales in both healthcare-associated and community-onset infections. The marked increase in CRO-NS *E. coli* observed in our cohort parallels global trends and underscores the need for ongoing surveillance, especially in community settings where empirical antimicrobial strategies remain challenging.^23,30–32^ While *K. pneumoniae* and *P. mirabilis* accounted for a minority of cases and did not demonstrate significant temporal increases, the non-significant upward trend in *K. oxytoca* prevalence warrants further monitoring.

While we performed an exploratory multivariable analysis to evaluate associations between demographic characteristics and CRO-NS bacteraemia, the primary contribution of this study lies in the longitudinal descriptive assessment of resistance trends. The annual prevalence estimates and accompanying temporal trends (Figure 1) provide the most clinically actionable insights, whereas regression results should be interpreted as supportive and descriptive, rather than as a comprehensive predictive risk model.

Despite the overall increase in CRO-NS, most established risk factors for Gram-negative bacteraemia remained relatively stable over the study period. However, we observed significant increases in the prevalence of moderate or severe liver disease, HIV, antibiotic exposure within 30 days, and use of penicillins within 90 days among patients with CRO-NS isolates. These observations are consistent with prior studies identifying immunocompromised status, chronic comorbidities, and recent antimicrobial exposure as key contributors to ESBL acquisition.^7,8,16,20,33–37^ The increasing burden of these risk factors may partially explain the observed rise in CRO-NS bacteraemia, broader influences, including environmental factors, international travel, changes in community antimicrobial use, and microbial ecology, likely also contribute.^38,39^

Although nine years represents a relatively modest observation period for detecting large structural shifts in epidemiologic risk factors, measurable changes in AMR trends have been documented over comparable time frames in cohort studies of ESBL-producing Enterobacterales. Importantly, our analyses were intended to be descriptive rather than causal, focusing on characterization of longitudinal patterns within our health system rather than identification of definitive drivers of resistance emergence.

Notably, many previous studies evaluating risk factors for ESBL-producing bacteraemia acquisition were conducted prior to the COVID-19 pandemic and predominantly among non-Black or African American populations.^23,40^ Our study is among the few to evaluate these trends in a predominantly Black or African American population, within a rural region of Southwest Georgia significantly impacted by healthcare disparities.^41^ Although the COVID-19 pandemic disrupted healthcare delivery and antimicrobial stewardship efforts globally, we found no significant changes in patient demographics or comorbidities between the pre- and post-pandemic periods. However, the increase in recent antibiotic use during the pandemic is consistent with reports of heightened empirical antimicrobial prescribing, particularly in regions like Southwest Georgia, which experienced disproportionate COVID-19 morbidity and mortality among Black communities.^42^ These findings suggest that increases in CRO-NS prevalence preceded the pandemic and reflect broader, pre-existing resistance trends.

We also observed that CRO-NS bacteraemia disproportionately affected older adults with multiple comorbidities and occurred more frequently among female patients, findings consistent with recent literature linking high disease burden to increased infection risk and healthcare exposure.^43^ However, in contrast to previous studies, neither sex nor older age was independently associated with CRO-NS detection in adjusted analyses.^8,33^ These results should therefore be interpreted cautiously, and our findings are best viewed as descriptive of the populations affected by CRO-NS bacteraemia rather than definitive assessments of independent risk.

Interestingly, Black or African American race was significantly associated with lower odds of CRO-NS bacteraemia, which diverges from prior studies reporting higher rates of AMR.^27^ Several factors may explain this discrepancy, including potential differences in healthcare-seeking behaviour, unmeasured confounders, and limitations in race categorization based on self-report at hospital admission. This finding warrants further investigation, particularly considering persistent racial health disparities in the U.S. South.

The steady rise in community-onset CRO-NS bacteraemia has important implications for empirical treatment strategies, antimicrobial stewardship, and public health interventions. The predominance of *E. coli* as the causative pathogen and the high proportion of urinary tract sources emphasize the need for targeted outpatient stewardship efforts, especially in individuals with known risk factors such as recent antibiotic exposure, advanced liver disease, or HIV. Moreover, the stable prevalence of many traditional risk factors emphasizes the complexity of resistance evolution and limits reliance on historical predictors alone when assessing patient risk in community settings.

## Limitations

Several limitations should be considered when interpreting our findings. First, this study was conducted in a single health system, which may limit generalizability. However, our health system serves both urban and rural populations.^42^ Notably, the majority of counties within its catchment area are classified as rural according to US Census Bureau urban–rural definitions, providing insight into AMR trends across diverse patient groups in Southwest Georgia.^44^ Second, the retrospective design introduces potential for missing data and unmeasured confounding, particularly regarding outpatient exposures and social determinants of health.

Third, our microbiological classification was based on ceftriaxone susceptibility, and we reported CRO-NS as the primary resistance phenotype. Although CRO-NS has been used as a surrogate for ESBL production in prior studies, including the MERINO trial, and is consistent with current IDSA guidance, we did not perform routine molecular confirmation of ESBL genes.^6,21,22^ As a result, we were unable to distinguish ESBL production from other mechanisms of CRO-NS. Routine phenotypic testing for ESBL production also remains controversial due to the potential for false positives or negatives, particularly when organisms co-produce multiple β-lactamase enzymes or when laboratory practices vary across institutions.^21^ Our institution uses MIC breakpoints to categorize isolates as susceptible, intermediate, or resistant, with only limited genetic testing for ESBL enzymes (e.g. CTX-M). It is therefore possible that the true burden of CRO-NS bacteraemia was underestimated during the earlier years of our study, particularly if updated breakpoints were not implemented uniformly or promptly within our institution.^45^ As a result, our findings may not fully capture the broader epidemiology of CRO-NS or ESBL-producing Enterobacterales in the community setting.

We also lacked access to medical records from outside facilities, which may have underestimated prior healthcare exposures or antimicrobial use not captured within our system.

Because cases were included on a per–calendar year basis and episode-to-episode intervals were not systematically captured, some recurrent infections recorded across adjacent years may have represented closely spaced clinical episodes rather than clearly distinct events. This approach may have introduced limited misclassification of recurrent bacteraemia frequency. However, given that our primary analyses focused on annual prevalence and temporal trends rather than patient-level recurrence rates, the impact on the main study findings is likely minimal.

In addition, the multivariable regression analysis should be interpreted as exploratory. Comprehensive clinical risk-factor data were available only for patients with CRO-NS bacteraemia and were not systematically captured for patients with ceftriaxone-susceptible isolates, which precluded inclusion of key established predictors such as prior ESBL infection, recent antimicrobial exposure, healthcare contact, and underlying comorbidities in comparative modelling. Accordingly, regression analyses were intentionally limited to demographic variables uniformly available across the cohort and were designed to provide adjusted descriptive context rather than to develop or validate a comprehensive risk prediction model for CRO-NS bacteraemia. Because comparator clinical risk-factor data were unavailable, we were unable to fully characterize differential risk profiles between patients with non-susceptible and susceptible infections.

### Conclusion

This 9-year longitudinal study demonstrated a significant and sustained increase in community-onset bacteraemia due to CRO-NS *E. coli*, *K. pneumoniae*, *K. oxytoca*, and *P. mirabilis*, with the burden more than doubling over time, driven primarily by predominantly by *E. coli*. While many traditional risk factors for Gram-negative bacteraemia remained stable, increasing rates of advanced liver disease, HIV, and recent antibiotic exposure reaffirm the clinical importance of these variables in resistance risk stratification and empiric therapy decisions. Exploratory regression revealed that Black or African American race was independently associated with lower odds of CRO-NS bacteraemia, whereas age and sex were not significantly were not independently predictive.

These findings emphasize the growing burden of AMR in community-associated infections and reinforce the need for ongoing surveillance, refined empiric prescribing practices, and targeted antimicrobial stewardship initiatives. Clinicians should maintain heightened awareness of CRO-NS and ESBL risk, even in community settings and among patients without recent healthcare exposure. Further research is needed to clarify drivers of resistance emergence and to develop predictive tools that integrate both patient-level and community-level factors to guide prevention and early intervention efforts.

## Supplementary Material

dlag014_Supplementary_Data

## Data Availability

The datasets generated and analysed during the current study are not publicly available due to institutional policies and patient privacy protections but are available from the corresponding author on reasonable request, contingent upon approval from the University of Georgia Institutional Review Board.

## Supplementary data

Tables S1 to S3 is available as Supplementary data at *JAC-AMR* Online.

## References

[1] Antimicrobial Resistance Collaborators . Global burden of bacterial antimicrobial resistance in 2019: a systematic analysis. Lancet 2022; 399: 629–55. 10.1016/S0140-6736(21)02724-0 35065702 PMC8841637

[2] Mcadam AJ . Enterobacteriaceae? Enterobacterales? What should we call enteric Gram-negative bacilli? A micro-comic strip. J Clin Microbiol 2020; 58: 10.1128/Jcm.01888-19. 10.1128/Jcm.01888-19 PMC6989070 31992653

[3] Santos AL, Dos Santos AP, Ito CRM et al Profile of enterobacteria resistant to beta-lactams. Antibiotics (Basel) 2020; 9: 410. 10.3390/Antibiotics9070410 32679663 PMC7400480

[4] Castanheira M, Simner PJ, Bradford PA. Extended-spectrum Β-lactamases: an update on their characteristics, epidemiology and detection. JAC Antimicrob Resist 2021; 3: Dlab092. 10.1093/Jacamr/Dlab092 34286272 PMC8284625

[5] Paterson DL, Bonomo RA. Extended-spectrum Β-lactamases: a clinical update. Clin Microbiol Rev 2005; 18: 657–86. 10.1128/CMR.18.4.657-686.2005 16223952 PMC1265908

[6] Tamma PD, Heil EL, Justo JA et al IDSA 2024 guidance on the treatment of antimicrobial resistant Gram-negative infections. Clin Infect Dis 2024; 10.1093/cid/ciae403. 39108079

[7] Kassakian SZ, Mermel LA. Changing epidemiology of infections due to extended spectrum beta-lactamase producing bacteria. Antimicrob Resist Infect Control 2014; 3: 9. 10.1186/2047-2994-3-9 24666610 PMC4230027

[8] Raphael E, Glymour MM, Chambers HF. Trends in prevalence of extended-spectrum beta-lactamase-producing *Escherichia coli* isolated from patients with community- and healthcare-associated bacteriuria: results from 2014 to 2020 in an urban safety-net healthcare system. Antimicrob Resist Infect Control 2021; 10: 118. 10.1186/S13756-021-00983-Y 34380549 PMC8359060

[9] CDC . COVID-19: U.S. impact on antimicrobial resistance, special report 2022. National Center For Emerging And Zoonotic Infectious Diseases, 2022.

[10] Benetazzo L, Delannoy PY, Houard M et al Combination therapy with aminoglycoside in bacteremiasdue to ESBL-producing enterobacteriaceae in ICU. Antibiotics 2020; 9: 777. 10.3390/Antibiotics9110777 33158238 PMC7694250

[11] Namikawa H, Imoto W, Yamada K et al Predictors of mortality from extended-spectrum beta-lactamase-producing Enterobacteriaceae bacteremia. Emerg Microbes Infect 2023; 12: 2217951. 10.1080/22221751.2023.2217951 37219067 PMC10243396

[12] Xiao T, Wu Z, Shi Q et al A retrospective analysis of risk factors and outcomes in patients with extended-spectrum beta-lactamase-producing *Escherichia coli* bloodstream infections. J Glob Antimicrob Resist 2019; 17: 147–56. 10.1016/J.Jgar.2018.12.014 30634054

[13] Marimuthu K, Ng OT, Cherng BPZ et al Antecedent carbapenem exposure as A risk factor for non-carbapenemase-producing carbapenem-resistant Enterobacteriaceae and carbapenemase-producing Enterobacteriaceae. Antimicrob Agents Chemother 2019; 63: E00845–19. 10.1128/AAC.00845-19 31383670 PMC6761519

[14] Moghnieh R, Abdallah D, Jadayel M et al Epidemiology, risk factors, and prediction score of carbapenem resistance among inpatients colonized or infected with 3rd generation cephalosporin resistant enterobacterales. Sci Rep 2021; 11: 14757. 10.1038/S41598-021-94295-1 34285312 PMC8292374

[15] Chen X, Zhou M, Yan Q et al Risk factors for carbapenem-resistant enterobacterales infection among hospitalized patients with previous colonization. J Clin Lab Anal 2022; 36: E24715. 10.1002/Jcla.24715 36181301 PMC9701893

[16] Augustine MR, Testerman TL, Justo JA et al Clinical risk score for prediction of extended-spectrum Β-lactamase–producing Enterobacteriaceae in bloodstream isolates. Infect Control Hosp Epidemiol 2017; 38: 266–72. 10.1017/Ice.2016.292 27989244

[17] Hayakawa K, Gattu S, Marchaim D et al Epidemiology and risk factors for isolation of Escherichia coli producing CTX-M-type extended-spectrum Β-lactamase in a large U.S. Medical Center. Antimicrob Agents Chemother 2013; 57: 4010–8. 10.1128/AAC.02516-12 23752516 PMC3719715

[18] Kaya O, Akcam FZ, Gonen I et al Risk factors for bacteremia due to extended-spectrum beta-lactamase-producing *Escherichia coli* in a Turkish Hospital. J Infect Dev Ctries 2013; 7: 507–12. 10.3855/Jidc.2788 23857384

[19] Nguyen ML, Toye B, Kanji S et al Risk factors for and outcomes of bacteremia caused by extended-spectrum β-lactamase–producing *Escherichia coli* and *Klebsiella* species at a Canadian Tertiary Care Hospital. Can J Hosp Pharm 2015; 68: 136–43. 10.4212/Cjhp.V68i2.1439 25964685 PMC4414075

[20] Tumbarello M, Trecarichi EM, Bassetti M et al Identifying patients harboring extended-spectrum-Β-lactamase-producing Enterobacteriaceae on hospital admission: derivation and validation of a scoring system. Antimicrob Agents Chemother 2011; 55: 3485–90. 10.1128/AAC.00009-11 21537020 PMC3122446

[21] Mathers AJ, Lewis JS. CON: testing for ESBL production is unnecessary for ceftriaxone-resistant enterobacterales. JAC Antimicrob Resist 2021; 3: Dlab020. 10.1093/Jacamr/Dlab020 34223109 PMC8210140

[22] Harris PNA, Tambyah PA, Lye DC et al Effect of piperacillin-tazobactam vs meropenem on 30-day mortality for patients with *E. coli* or *Klebsiella pneumoniae* bloodstream infection and ceftriaxone resistance: a randomized clinical trial. JAMA 2018; 320: 984–94. 10.1001/Jama.2018.12163 30208454 PMC6143100

[23] Ince D, Fiawoo S, Choudhury R et al Epidemiology of Gram-negative bloodstream infections in the United States: results from a cohort of 24 hospitals. Open Forum Infect Dis 2023; 10: Ofad265. 10.1093/Ofid/Ofad265 37465379 PMC10350481

[24] Harris PA, Taylor R, Thielke R et al Research electronic data capture (Redcap)—a metadata-driven methodology and workflow process for providing translational research informatics support. J Biomed Inform 2009; 42: 377–81. 10.1016/J.Jbi.2008.08.010 18929686 PMC2700030

[25] Harris PA, Taylor R, Minor BL et al The Redcap Consortium: building an International Community of Software Platform Partners. J Biomed Inform 2019; 95: 103208. 10.1016/J.Jbi.2019.103208 31078660 PMC7254481

[26] Charlson ME, Pompei P, Ales KL et al A new method of classifying prognostic comorbidity in longitudinal studies: development and validation. J Chronic Dis 1987; 40: 373–83. 10.1016/0021-9681(87)90171-8 3558716

[27] Hemenway AN, Biagi M, Murrey TF et al Association of race or ethnicity with extended-spectrum beta-lactamase production in *Escherichia coli*: a case control study. Open Forum Infect Dis 2024; 11: Ofae516. 10.1093/Ofid/Ofae516 39391100 PMC11465405

[28] Atamna A, Babich T, Margalit I et al Does accepted definition of *Clostridioides difficile* infection (CDI) severity predict poor outcomes in older adults? Aging Clin Exp Res 2022; 34: 583–9. 10.1007/S40520-021-01953-5 34426944

[29] CDC . Older Adults. Chronic Disease Indicators. Https://Www.Cdc.Gov/Cdi/Indicator-Definitions/Older-Adults.Html.

[30] Bezabih YM, Sabiiti W, Alamneh E et al The global prevalence and trend of human intestinal carriage of ESBL-producing *Escherichia coli* in the community. J Antimicrob Chemother 2021; 76: 22–9. 10.1093/Jac/Dkaa399 33305801

[31] Mcdanel J, Schweizer M, Crabb V et al Incidence of extended-spectrum Β-lactamase (ESBL)-producing *Escherichia coli* and *Klebsiella* infections in the United States: a systematic literature review. Infect Control Hosp Epidemiol 2017; 38: 1209–15. 10.1017/Ice.2017.156 28758612

[32] Smith MW, Carrel M, Shi Q et al Spatiotemporal distribution of community-acquired phenotypic extended-spectrum beta-lactamase *Escherichia coli* in United States counties, 2010–2019. Infect Control Hosp Epidemiol 2024; 45: 540–2. 10.1017/Ice.2023.266 38073591 PMC11007319

[33] Ben-Ami R, Rodríguez-Baño J, Arslan H et al A multinational survey of risk factors for infection with extended-spectrum Β-lactamase-producing Enterobacteriaceae in nonhospitalized patients. Clin Infect Dis 2009; 49: 682–90. 10.1086/604713 19622043

[34] Halldórsdóttir AM, Hrafnkelsson B, Einarsdóttir K et al Prevalence and risk factors of extended-spectrum beta-lactamase producing *E. coli* causing urinary tract infections in Iceland during 2012–2021. Eur J Clin Microbiol Infect Dis 2024; 43: 1689–97. 10.1007/S10096-024-04882-Z 38935227 PMC11349795

[35] Johnson SW, Anderson DJ, May DB et al Utility of a clinical risk factor scoring model in predicting infection with extended-spectrum Β-lactamase-producing Enterobacteriaceae on hospital admission. Infect Control Hosp Epidemiol 2013; 34: 385–92. 10.1086/669858 23466912 PMC3641565

[36] Laupland KB, Church DL, Vidakovich J et al Community-onset extended-spectrum Β-lactamase (ESBL) producing *Escherichia coli*: importance of international travel. J Infect 2008; 57: 441–8. 10.1016/J.Jinf.2008.09.034 18990451

[37] Peña C, Gudiol C, Tubau F et al Risk-factors for acquisition of extended-spectrum Β-lactamase-producing *Escherichia coli* among hospitalised patients. Clin Microbiol Infect 2006; 12: 279–84. 10.1111/J.1469-0691.2005.01358.X 16451416

[38] Bengtsson-Palme J, Kristiansson E, Larsson DGJ. Environmental factors influencing the development and spread of antibiotic resistance. FEMS Microbiol Rev 2018; 42: Fux053. 10.1093/Femsre/Fux053 29069382 PMC5812547

[39] Günther T, Kramer-Schadt S, Fuhrmann M et al Environmental factors associated with the prevalence of ESBL/AmpC-producing *Escherichia coli* in wild boar (*Sus scrofa*). Front Vet Sci 2022; 9: 980554. 10.3389/Fvets.2022.980554 36311652 PMC9608181

[40] Goodman KE, Lessler J, Cosgrove SE et al A clinical decision tree to predict whether a bacteremic patient is infected with an extended-spectrum Β-lactamase–producing organism. Clin Infect Dis 2016; 63: 896–903. 10.1093/Cid/Ciw425 27358356 PMC5019284

[41] Byrnes H, Suneson G. This is Where Georgia’s Health Care System Ranks in the US | Georgia. Thecentersquare.Com. Https://Www.Thecentersquare.Com/Georgia/This-Is-Where-Georgia-S-Health-Care-System-Ranks-In-The-Us/Article_8a754736-Cccb-530f-B02c-A3f3c58ef294.Html.

[42] Chastain DB, Osae SP, Thomas GM et al Clinical severity on hospital admission for COVID-19: an analysis of social determinants of health from an early hot spot in the Southeastern U.S. J Prim Care Community Health 2022; 13: 21501319221092244. 10.1177/21501319221092244 35426348 PMC9016530

[43] Theodorakis N, Feretzakis G, Hitas C et al Immunosenescence: how aging increases susceptibility to bacterial infections and virulence factors. Microorganisms 2024; 12: 2052. 10.3390/Microorganisms12102052 39458361 PMC11510421

[44] Urban And Rural. https://www.census.gov/programs-surveys/geography/guidance/geo-areas/urban-rural.html.

[45] Prinzi A . Extended-Spectrum Beta-Lactamases: To Confirm Or Not Confirm? ASM.Org. Https://Asm.Org:443/Articles/2022/April/Extended-Spectrum-Beta-Lactamases-To-Confirm-Or-No

