# Supplementary material for: Longitudinal trends in community-onset bacteraemia caused by ceftriaxone non-susceptible Escherichia coli, Proteus mirabilis, Klebsiella oxytoca, and Klebsiella pneumoniae (2016–2024)
Source: JAC Antimicrob Resist. 2026 Feb 13;8(1):dlag014. doi: 10.1093/jacamr/dlag014 (PMC12900576; doi:10.1093/jacamr/dlag014)
Supplement: dlag014_Supplementary_Data [file dlag014_supplementary_data.docx]

**Supplemental Table 1:** Prevalence of Community-Onset Ceftriaxone Non-Susceptible *E. coli, K. pneumoniae, K. oxytoca*, and *P. mirabilis* Bacteremia from 2016 to 2024

|  | 2016 | 2017 | 2018 | 2019 | 2020 | 2021 | 2022 | 2023 | 2024 | Overall Prevalence  2016-2024 | Annual change | p-values |
| --- | --- | --- | --- | --- | --- | --- | --- | --- | --- | --- | --- | --- |
|  |  |  |  |  |  |  |  |  |  |  |  |  |
| **Annual Prevalence n (%)** | 9 (6.8%) | 8 (5.3%) | 12 (6.9%) | 16 (9.8%) | 12 (6.5%) | 18 (12.0%) | 12 (8.3%) | 18 (11.8%) | 22 (15.5%) | 127  (9.1%) | 0.99% | 0.008 |
| **Species,** **n (%)** |  |  |  |  |  |  |  |  |  |  |  |  |
| *Escherichia coli* | 6 (6.2%) | 8 (8.5%) | 11 (8.9%) | 11 (10.7%) | 9 (7.0%) | 16 (17.2%) | 11 (12.9%) | 16 (16.5%) | 18 (21.2%) | 106 (11.7%) | 1.64% | 0.002 |
| *Klebsiella pneumoniae* | 1 (20.0%) | 0 (0.0%) | 0 (0.0%) | 1 (50.0%) | 2 (66.7%) | 0 (0.0%) | 0 (0.0%) | 0 (0.0%) | 0 (0.0%) | 4  (14.8%) | -2.16% | 0.550 |
| *Klebsiella oxytoca* | 2 (9.1%) | 0 (0.0%) | 1 (2.9%) | 3 (7.0%) | 0 (0.0%) | 2 (50.0%) | 1 (2.7%) | 2 (6.1%) | 4 (10.8%) | 15  (4.6%) | 1.13% | 0.608 |
| *Proteus mirabilis* | 0 (0.0%) | 0 (0.0%) | 0 (0.0%) | 1 (6.7%) | 1 (9.1%) | 0 (0.0%) | 0 (0.0%) | 0 (0.0%) | 0 (0.0%) | 2  (1.5%) | -0.11% | 0.825 |

Prevalence was calculated annually as the proportion of ceftriaxone non-susceptible isolates relative to the total number of isolates (Ceftriaxone non-susceptible and ceftriaxone susceptible) for each species

**Supplemental Table 2:**  Baseline Characteristics Adults with Community-Onset Ceftriaxone Non-Susceptible *E. coli, K. pneumoniae, K. oxytoca*, and *P. mirabilis* Bacteremia Per Year

| Characteristic | 2016 (n=9) | 2017 (n=8) | 2018 (n=12) | 2019 (n=16) | 2020 (n=12) | 2021 (n=18) | 2022 (n=12) | 2023 (n=18) | 2024 (n=22) | Overall (n=127) | Annual Change | P-value |
| --- | --- | --- | --- | --- | --- | --- | --- | --- | --- | --- | --- | --- |
| Age (years), median (IQR) | 72  (60, 81) | 63  (55, 76) | 71  (59, 78) | 72  (60, 76) | 65  (54, 70) | 68  (64, 74) | 61  (57, 68) | 64  (57, 73) | 77  (58, 81) | 67  (58, 77) | -0.05 | 0.946 |
| Female sex | 56% | 75% | 75% | 44% | 58% | 44% | 42% | 78% | 59% | 57% | -0.73% | 0.718 |
| Race |  |  |  |  |  |  |  |  |  |  |  |  |
| - White | 44% | 50% | 42% | 38% | 58% | 39% | 50% | 39% | 41% | 43% | -0.49% | 0.619 |
| - Black or African American | 56% | 50% | 50% | 63% | 42% | 61% | 42% | 50% | 55% | 53% | -0.37% | 0.722 |
| - Other | 0% | 0% | 8% | 0% | 0% | 0% | 8% | 11% | 5% | 4% | 8.56% | 0.157 |
| Admitting Source |  |  |  |  |  |  |  |  |  |  |  |  |
| - Home | 67% | 75% | 83% | 81% | 67% | 72% | 83% | 67% | 91% | 77% | 1.05% | 0.397 |
| - Long term care | 33% | 25% | 17% | 19% | 33% | 22% | 0% | 28% | 9% | 20% | -1.98% | 0.181 |
| - Other *^a^* | 0% | 0% | 0% | 0% | 0% | 6% | 17% | 6% | 0% | 3% | 0.93% | 0.223 |
| Infection source |  |  |  |  |  |  |  |  |  |  |  |  |
| - Urinary tract | 33% | 75% | 92% | 56% | 75% | 50% | 75% | 67% | 77% | 67% | 1.85% | 0.449 |
| - Respiratory tract | 0% | 0% | 0% | 6% | 0% | 0% | 0% | 6% | 5% | 2% | 0.48% | 0.197 |
| - Skin and Soft tissue | 11% | 0% | 0% | 13% | 17% | 6% | 0% | 6% | 0% | 6% | -0.58% | 0.523 |
| - Gastrointestinal tract | 22% | 13% | 0% | 6% | 0% | 22% | 17% | 11% | 13% | 12% | 0.17% | 0.888 |
| - Unknown *^b^* | 33% | 13% | 8% | 19% | 8% | 22% | 8% | 11% | 5% | 13% | -1.93% | 0.099 |
| History of ESBL within 1 year, any source | 33% | 13% | 8% | 0% | 42% | 17% | 17% | 11% | 5% | 14% | -1.43% | 0.446 |
| Comorbid conditions and medical history |  |  |  |  |  |  |  |  |  |  |  |  |
| - Gastrointestinal Procedures within 30 days | 0% | 0% | 8% | 13% | 0% | 17% | 0% | 0% | 9% | 6% | 0.40% | 0.668 |
| - Urological Procedures within 30 days | 0% | 25% | 8% | 0% | 17% | 0% | 8% | 11% | 0% | 6% | -0.69% | 0.579 |
| - Injection Drug Use | 0% | 0% | 0% | 0% | 0% | 0% | 0% | 6% | 0% | <1% | - | - |
| - Congestive heart failure | 33% | 25% | 17% | 18% | 33% | 33% | 42% | 22% | 18% | 26% | -0.07% | 0.954 |
| - Chronic pulmonary disease | 2% | 13% | 17% | 13% | 17% | 22% | 17% | 11% | 23% | 17% | 0.13% | 0.845 |
| - Diabetes | 56% | 38% | 33% | 25% | 50% | 61% | 42% | 50% | 36% | 43% | 0.23% | 0.891 |
| - Moderate or severe renal disease | 22% | 0% | 8% | 18% | 8% | 17% | 17% | 11% | 0% | 11% | -0.68% | 0.544 |
| - Moderate or severe liver disease | 0% | 0% | 0% | 6% | 8% | 6% | 17% | 11% | 5% | 6% | 1.40% | 0.045 |
| - Metastatic solid tumor | 0% | 0% | 0% | 13% | 0% | 17% | 8% | 0% | 9% | 6% | 0.95% | 0.290 |
| - Solid tumor (non- metastatic) | 33% | 25% | 17% | 25% | 0% | 28% | 0% | 0% | 18% | 16% | -2.77% | 0.103 |
| - HIV | 0% | 0% | 0% | 0% | 0% | 6% | 0% | 6% | 5% | 2% | 0.67% | 0.035 |
| Charleson Comorbidity Index, median (IQR) | 6  (5, 7) | 4  (2, 6) | 4  (3, 5) | 6  (4, 7) | 4  (2, 5) | 8  (4, 9) | 5  (3, 6) | 5  (4, 6) | 6  (4, 8) | 5  (3, 7) | 0.08 | 0.652 |
| Medication use within 90 days |  |  |  |  |  |  |  |  |  |  |  |  |
| - Glucocorticoids | 33% | 13% | 0% | 13% | 25% | 11% | 17% | 17% | 18% | 18% | -0.27% | 0.838 |
| - Proton Pump Inhibitors | 67% | 38% | 42% | 44% | 25% | 28% | 33% | 33% | 41% | 38% | -2.47% | 0.119 |
| - Chemotherapy | 11% | 0% | 0% | 13% | 8% | 17% | 17% | 11% | 14% | 11% | 1.34% | 0.095 |
| Antibiotic use within 30 days *^c^* | 22% | 25% | 33% | 25% | 50% | 33% | 25% | 44% | 55% | 37% | 2.98% | 0.044 |
| - Anti-pseudomonal*^d^* | 0% | 100% | 50% | 25% | 17% | 50% | 67% | 38% | 25% | 36% | -0.48% | 0.909 |
| - Anti-MRSA*^e^* | 0% | 0% | 100% | 0% | 17% | 33% | 3% | 25% | 17% | 25% | 0.69% | 0.876 |
| - Cephalosporin *^f^* | 100% | 0% | 25% | 0% | 67% | 17% | 33% | 38% | 50% | 38% | -0.90% | 0.845 |
| - Fluoroquinolone | 0% | 50% | 50% | 25% | 17% | 50% | 67% | 38% | 17% | 32% | 1.46% | 0.631 |
| - Penicillins *^g^* | 0% | 0% | 0% | 25% | 0% | 0% | 0% | 13% | 25% | 11% | 1.88% | 0.206 |
| - Carbapenems | 0% | 0% | 0% | 0% | 0% | 0% | 33% | 13% | 0% | 4% | 1.73% | 0.262 |
| - Other *^h^* | 0% | 50% | 0% | 50% | 50% | 33% | 33% | 0% | 50% | 32% | 1.67% | 0.613 |
| Antibiotic use within 90 days *^c^* | 78% | 38% | 58% | 63% | 67% | 56% | 33% | 67% | 77% | 61% | 0.47% | 0.830 |
| - Anti-pseudomonal*^d^* | 57% | 100% | 71% | 32% | 38% | 70% | 75% | 42% | 71% | 62% | -1.25% | 0.685 |
| - Anti-MRSA*^e^* | 57% | 0% | 71% | 21% | 25% | 40% | 0% | 25% | 35% | 37% | -2.26% | 0.496 |
| - Cephalosporin *^f^* | 71% | 0% | 43% | 26% | 75% | 50% | 50% | 67% | 59% | 56% | 3.13% | 0.341 |
| - Fluoroquinolone | 57% | 67% | 57% | 11% | 13% | 60% | 75% | 33% | 53% | 45% | -0.52% | 0.873 |
| - Penicillins *^g^* | 0% | 0% | 0% | 5% | 0% | 0% | 0% | 17% | 29% | 10% | 2.70% | 0.033 |
| - Carbapenems | 0% | 33% | 0% | 11% | 13% | 0% | 25% | 8% | 12% | 10% | 0.19% | 0.907 |
| - Other *^h^* | 0% | 33% | 14% | 11% | 50% | 20% | 50% | 25% | 53% | 31% | 4.45% | 0.066 |
| Hospitalization within 90 days | 67% | 13% | 33% | 56% | 50% | 28% | 25% | 22% | 55% | 39% | -1.08% | 0.687 |
| Hospitalization within 1 year | 89% | 25% | 50% | 75% | 50% | 56% | 42% | 56% | 82% | 61% | 0.45% | 0.875 |

*^a^*  Defined as being admitted from shelters, rehabilitation centers, or correctional facility.

*^b^* Unclear source of infection or infection was due to multiple sources

*^c^* Antibiotics may be classified under more than one pharmacological drug class

*^d^* Anti-pseudomonal includes Cefepime, Pip-Tazo, Meropenem, Gentamicin, Ciprofloxacin, Levofloxacin

*^e^* Anti-MRSA includes Vancomycin, TMP-SMX, Doxycycline, Clindamycin

*^f^* Cephalosporin includes Cefazolin, Cefdinir, Cefepime, Cefoxitin, Ceftriaxone, Cephalexin

*^g^* Penicillins includes Amoxicillin, Amox-Clav, Ampicillin, Amp-Sulb

*^h^* Other includers Azithromycin, Clarithromycin, Metronidazole, Nitrofurantoin

**Supplemental Table 3** Baseline Characteristics for Adults with Community-Onset Ceftriaxone Non-Susceptible *E. coli, K. pneumoniae, K. oxytoca*, and *P. mirabilis* Bacteremia During the Pre-COVID and Post-COVID Periods

| Characteristic | Pre-COVID  n=45 | Post-COVID  n=82 | P-value |
| --- | --- | --- | --- |
| Age (years), median (IQR) | 71  (60, 79) | 66.5  (58, 76.75) | 0.382 |
| Female sex | 56% | 57% | 0.996 |
| Race |  |  |  |
| - White | 42% | 44% | 1 |
| - Black or African American | 56% | 51% | 0.777 |
| - Other | 2% | 5% | 0.795 |
| Admitting Source |  |  |  |
| - Home | 78% | 77% | 1 |
| - Long term care | 22% | 18% | 0.765 |
| - Other*^a^* | 0% | 5% | 0.329 |
| Infection Source |  |  |  |
| - Urinary Tract | 64% | 68% | 0.807 |
| - Respiratory | 2% | 2% | 1 |
| - Skin and Soft Tissue | 7% | 5% | 0.987 |
| - Gastrointestinal | 9% | 13% | 0.639 |
| - Other/Unknown*^b^* | 18% | 11% | 0.421 |
| History of ESBL within 1 year, any source, n (%) | 11% | 16% | 0.202 |
| Comorbid conditions and medical history |  |  |  |
| - Recent Gastrointestinal Procedures within 30 days | 7% | 6% | 1 |
| - Recent Urological Procedures within 30 days | 7% | 6% | 1 |
| - Injection Drug Use | 0% | 1% | 1 |
| - Congestive heart failure (CHF) | 22% | 28% | 0.613 |
| - Chronic pulmonary disease | 16% | 18% | 0.885 |
| - Diabetes | 36% | 48% | 0.263 |
| - Moderate or severe renal disease | 13% | 10% | 0.749 |
| - Moderate or severe liver disease | 2% | 9% | 0.308 |
| - Solid tumor (non metastatic) | 24% | 11% | 0.082 |
| - Metastatic solid tumor | 4% | 7% |  |
| - HIV | 0% | 4% | 0.491 |
| - Charleson Comorbidity Index | 5  (3, 7) | 5  (3, 7) | 0.313 |
| Medication use within 90 days |  |  |  |
| - Glucocorticoids | 13% | 21% | 0.427 |
| - Proton pump inhibitors | 47% | 33% | 0.181 |
| - Chemotherapy | 7% | 13% | 0.387 |
| Antibiotic use within 30 days *^c^* | 27% | 43% | 0.110 |
| - Anti-pseudomonal*^d^* | 42% | 34% | 0.912 |
| - Anti-MRSA*^e^* | 33% | 23% | 0.737 |
| - Cephalosporin *^f^* | 25% | 43% | 0.451 |
| - Fluoroquinolone | 33% | 31% | 1 |
| - Penicillins *^g^* | 8% | 11% | 1 |
| - Carbapenems | 0% | 6% | 0.985 |
| - Other *^h^* | 25% | 34% | 0.812 |
| Antibiotic use within 90 days *^c^* | 60% | 62% | 0.951 |
| - Anti-pseudomonal*^d^* | 67% | 59% | 0.665 |
| - Anti-MRSA*^e^* | 48% | 31% | 0.225 |
| - Cephalosporin *^f^* | 48% | 61% | 0.406 |
| - Fluoroquinolone | 44% | 45% | 1 |
| - Penicillins *^g^* | 4% | 14% | 0.319 |
| - Carbapenems | 11% | 10% | 1 |
| - Other *^h^* | 15% | 39% | 0.049 |
| Hospitalization within 90 days | 44% | 37% | 0.498 |
| Hospitalization within 360 days | 62% | 60% | 0.934 |

*^a^*  Defined as being admitted from shelters, rehabilitation centers, or correctional facility.

*^b^* Unclear source of infection or infection was due to multiple sources

*^c^* Antibiotics may be classified under more than one pharmacological drug class

*^d^* Anti-pseudomonal includes Cefepime, Pip-Tazo, Meropenem, Gentamicin, Ciprofloxacin, Levofloxacin

*^e^* Anti-MRSA includes Vancomycin, TMP-SMX, Doxycycline, Clindamycin

*^f^* Cephalosporin includes Cefazolin, Cefdinir, Cefepime, Cefoxitin, Ceftriaxone, Cephalexin

*^g^* Penicillins includes Amoxicillin, Amox-Clav, Ampicillin, Amp-Sulb

*^h^* Other includers Azithromycin, Clarithromycin, Metronidazole, Nitrofurantoin
